# SCCPMD: Probability matrix decomposition method subject to corrected similarity constraints for inferring long non-coding RNA–disease associations

**DOI:** 10.3389/fmicb.2022.1093615

**Published:** 2023-01-11

**Authors:** Lieqing Lin, Ruibin Chen, Yinting Zhu, Weijie Xie, Huaiguo Jing, Langcheng Chen, Minqing Zou

**Affiliations:** ^1^Center of Campus Network & Modern Educational Technology, Guangdong University of Technology, Guangzhou, China; ^2^School of Computer, Guangdong University of Technology, Guangzhou, China; ^3^Sports Department, Guangdong University of Technology, Guangzhou, China; ^4^Department of Experiment Teaching, Guangdong University of Technology, Guangzhou, China

**Keywords:** lncRNA-long noncoding RNA, disease, similarity correction, constraint probability matrix decomposition, associations prediction

## Abstract

Accumulating evidence has demonstrated various associations of long non-coding RNAs (lncRNAs) with human diseases, such as abnormal expression due to microbial influences that cause disease. Gaining a deeper understanding of lncRNA–disease associations is essential for disease diagnosis, treatment, and prevention. In recent years, many matrix decomposition methods have also been used to predict potential lncRNA-disease associations. However, these methods do not consider the use of microbe-disease association information to enrich disease similarity, and also do not make more use of similarity information in the decomposition process. To address these issues, we here propose a correction-based similarity-constrained probability matrix decomposition method (SCCPMD) to predict lncRNA–disease associations. The microbe-disease associations are first used to enrich the disease semantic similarity matrix, and then the logistic function is used to correct the lncRNA and disease similarity matrix, and then these two corrected similarity matrices are added to the probability matrix decomposition as constraints to finally predict the potential lncRNA–disease associations. The experimental results show that SCCPMD outperforms the five advanced comparison algorithms. In addition, SCCPMD demonstrated excellent prediction performance in a case study for breast cancer, lung cancer, and renal cell carcinoma, with prediction accuracy reaching 80, 100, and 100%, respectively. Therefore, SCCPMD shows excellent predictive performance in identifying unknown lncRNA–disease associations.

## Introduction

Non-coding RNAs such as microRNAs (miRNAs), Circular RNA (circRNA) and long non-coding RNAs (lncRNAs) play crucial roles in controlling the biological processes of plants and animals (Zhang et al., 2020b; Wang et al., 2021a, 2022). Owing to their roles as genetic regulators in the development of complex disorders such as cancer, miRNAs have the potential to serve as diagnostic markers and therapeutic targets (Chen et al., 2019b; Hill and Tran, 2021; Huang et al., 2022a,b). Several algorithmic models have also been developed for the exploration of miRNA–disease and miRNA-disease associations (Chen et al., 2019c, 2021a; Zhang et al., 2021a,b). However, as medicine advances, more and more studies have also shown that lncRNAs play an important role in many different diseases (Cao et al., 2019). LncRNAs are RNA molecules with transcriptional lengths above 200 nucleotides that lack protein-coding capabilities (Xing et al., 2021). For example, *HOXA-AS2* was identified as a novel cancer-associated lncRNA, which exhibits aberrant expression in a variety of malignancies, including breast, gastric, gallbladder, hepatocellular, and pancreatic cancers (Wang et al., 2018a). With increasing recognition of the importance of lncRNAs, more in-depth research has focused on the relationship between lncRNAs and diseases. However, traditional biological validation experiments are time-consuming and costly; thus, there is an urgent need to develop accurate and effective computational methods to determine possible lncRNA–disease associations. Many computational models have recently been developed to successfully predict possible lncRNA–disease associations, which can be classified into three main categories.

The first category is characterized by machine-learning methods (Zhang et al., 2020a; Lan et al., 2022). Chen and Yan (2013) proposed the first such approach to predict lncRNA–disease associations using Laplace regularized least squares in a semi-supervised learning framework. Subsequently, by combining genomic, glomerular, and transcriptomic data, Zhao et al. (2015) devised a computational method based on a simple Bayesian classifier approach, which led to the discovery of 707 potential cancer-associated lncRNAs. Zhu et al. (2021) predicted lncRNA–disease associations by integrating several similarity matrices and combining incremental principal component analysis and random forest techniques. However, supervised learning-based models such as support vector machine and plain Bayesian classifiers rely heavily on difficult-to-obtain negative sample (Chen et al., 2017).

The second category is based on building biological networks to predict lncRNA–disease associations (Zhang et al., 2019a, 2020c). Sun et al. (2014) proposed RWRlncD, a global network computational strategy that applies restart random wandering (RWR) on lncRNA functional similarity networks to infer potential associations between human lncRNAs and disease. Zhang et al. (2019b) integrated known topological interactions of lncRNA–disease, lncRNA–miRNA, and miRNA–disease to construct a linked tripartite network, and used the topology of the obtained network to calculate the similarity of disease pairs and lncRNA pairs. Finally, rule-based inference methods were used to predict new lncRNA–disease associations. Zhou et al. (2021) employed a rotating forest classifier to train prediction models after creating a heterogeneous network by combining relationships among miRNAs, lncRNAs, proteins, drugs, and diseases. However, the heterogeneous networks constructed by these network-based approaches relying on the relationships of lncRNAs themselves, miRNAs, proteins, and drugs to lncRNAs (diseases) can result in failure in reliable predictions of new diseases and/or new lncRNAs.

The third category includes matrix decomposition methods (Chen et al., 2018a,b, 2021b; Xie et al., 2021). To effectively predict probable relationships, Fu et al. (2018) employed matrix triple decomposition to split a data matrix from heterogeneous data sources into low-rank matrices and reconstruct the lncRNA–disease association matrix. Based on probabilistic matrix decomposition, Xuan et al. (2019) deduced probable lncRNA–disease associations by assuming that low-rank matrices are positively distributed with Gaussian noise. To enhance the potential association between lncRNAs and diseases, Gao et al. (2021) optimized the lncRNA and disease space by multi-labeling and fusing these labels. Finally, co-matrix decomposition was used to predict lncRNA–disease correlations. Wang et al. (2021b) treated the discovery of disease-associated lncRNA as a recommender system problem, and predicted the relationships between lncRNA and diseases using a graph-regularized non-negative matrix decomposition approach. (Liu et al., 2021) proposed an lncRNA–disease association prediction approach based on double sparse collaborative matrix decomposition. To boost the sparsity, the L2,1-norm was introduced to the conventional co-matrix decomposition method. However, none of the algorithms presented above use similar information of lncRNA and disease as constraints to optimize the matrix decomposition algorithm. Thus, there is still some room for improvement in the prediction performance.

Traditional probabilistic matrix decomposition only uses probabilistic linear models with Gaussian noise to model the interaction of lncRNAs with diseases. Based on the assumption that similar lncRNAs/diseases are usually interrelated with the corresponding disease/lncRNA, we here propose a correction-based similarity-constrained probability matrix decomposition (SCCPMD) method for predicting lncRNA–disease associations. Considering the noise effect of the similarity matrix of lncRNAs and diseases, the noise is reduced by correcting the similarity matrix using a logistic function to highlight strong correlations within the similarity range [0,1] while diluting weak correlations. The lncRNA and disease similarity are then used as constraints in the probability matrix decomposition process, resulting in two low-rank matrices to predict the potential lncRNA–disease association. Leave-one-out cross-validation (LOOCV) and five-fold cross-validation (5-fold CV) were performed to validate the predictive performance of SCCPMD using known lncRNA–disease association datasets. The final area under the curve (AUC) values of SCCPMD reached 0.9787 and 0.9528 ± 0.0036 with LOOCV and 5-fold CV, respectively, which were both better than the prediction performances obtained with existing advanced algorithms. In addition, we confirmed the effectiveness of SCCPMD in application to three test cases of human diseases: breast cancer, lung cancer, and renal cell carcinoma (RCC).

## Materials and methods

### Datasets

We used the LncRNADisease database (Bao et al., 2019), which provides a dataset of lncRNA–disease associations. After removing duplicate lncRNAs and diseases as well as non-human data, 1,690 unique experimentally validated lncRNA–disease associations were obtained, including 447 unique lncRNAs and 218 unique diseases. The lncRNA–disease associations were described by building a disease–lncRNA adjacency matrix, $Y\in R^{nl\times nd}$, where nl and nd represent the number of lncRNAs and diseases, respectively. The matrix Y is defined as follows:


(1) $$Y\left(i,j\right)=\{\begin{array}{l} 0\;\;\mathrm{lncRNA}\;l\left(i\right)\;\mathrm{has}\;\mathrm{no}\;\mathrm{association with disease}\;d\left(j\right)\;\\ 1\;\;\mathrm{lncRNA}\;l\left(i\right)\;\mathrm{is associated with disease}\;d\left(j\right) \end{array}$$

In other words, if an lncRNA $l_{i}$ is confirmed to be associated with a disease $d_{j}$, then Y(i,j) is set to 1; otherwise, Y(i,j) is 0.

### Semantic similarity of disease

We built a directed acyclic graph (DAG) based on the descriptor data from the Medical Subject Headings (MeSH) of the National Library of Medicine^1^ to determine the semantic similarity among diseases. A disease d is described by DAG(d)=(d,V(d),E(d)), where V(d) and E(d) are the vertex set and edge set of the DAG, respectively. Based on the DAG layer structure of disease d, we can calculate the semantic value (S) of disease m to disease d as follows:


(2) $$T_{d}\left(m\right)=\{\begin{array}{c} 1\;\;,\;\;\;if\;m=d\\ \max\{0.5^{*}T_{d}\left(m^{\prime }\right)|m^{\prime }\in childrenof\;m,if\;m\neq d \end{array}$$

According to the DAG of a disease, the semantic value of a disease is defined as the sum of the ancestral nodes of the disease and the semantic contribution value of the disease to itself, expressed by the following equation:


(3) $$T_{d}=\underset{m\in V\left(d\right)}{\sum }T_{d}\left(m\right)$$

Based on the above steps, we can construct the semantic similarity matrix SS to represent the semantic similarity between disease $d_{i}$ and disease $d_{j}$:


(4) $$SS\left(d_{i},d_{j}\right)=\frac{\sum_{m\in V\left(d_{i}\right)\cap V\left(d_{j}\right)}\left(T_{d_{i}}\left(m\right)+T_{d_{j}}\left(m\right)\right)}{T_{d_{i}}+T_{d_{j}}}$$

### Gaussian interaction profile kernel similarity for diseases

To address the sparsity of the semantic similarity matrix of diseases and integrate more information on disease similarity, we used microbe-disease associations to calculate Gaussian similarity of diseases. We downloaded human microbe-disease associations from the Human Microbe-Disease Association Database (HMDAD). Microbe-disease associations were described by creating a microbe-disease adjacency matrix, $A\in R^{m\times n}$, where m and d represent the number of microbes and diseases, respectively. As a measure of disease similarity, we constructed Gaussian interaction spectral kernel similarity using radial basis functions. We calculated the Gaussian interaction distribution based on the adjacency matrix A. The Gaussian interaction spectral kernel similarity between disease *d_i_* and disease *d_j_* can be calculated by the following equation:


(5) $$GD\left(d_{i},d_{j}\right)=\exp\left(-\gamma_{d}{\left|\left|A\left(:,i\right)-A\left(:,j\right)\right|\right|}^{2}\right)$$

(6) $$\gamma_{d}=\gamma /\left(\frac{1}{n}\overset{n}{\underset{i=1}{\sum }}{\left\|A\left(:,i\right)\right\|}^{2}\right)$$

### Integrated similarity for diseases

We combine the disease semantic similarity SS with the disease Gaussian similarity GD to construct the final disease similarity matrix SD. as follows, for disease $d_{i}$ and disease $d_{j}$, $SD\left(d_{i},d_{j}\right)=GD\left(d_{i},d_{j}\right)$ if SS=0 and $SD\left(d_{i},d_{j}\right)=SS\left(d_{i},d_{j}\right)$ otherwise.


(7) $$DS\left(d_{i},d_{j}\right)=\{\begin{array}{l} GD\left(d_{i},d_{j}\right)if\;\;SS\left(d_{i},d_{j}\right)=0\\ SS\left(d_{i},d_{j}\right)\;otherwise \end{array}$$

### Expression similarity of LncRNAs

LncRNA expression profiles can be utilized to reflect the similarity between lncRNAs, since related lncRNAs exhibit co-expression characteristics in various tissues (Chen et al., 2019a). For this purpose, we used RNA-sequencing data retrieved from the ArrayExpress database to create lncRNA expression profiles. The Spearman correlation coefficient between the expression profiles of two lncRNAs was then used to determine the degree of similarity in their expression patterns, defined as ES, where $ES\left(l_{i},l_{j}\right)\in \left[0,1\right]$ denotes the expression similarity of lncRNAs $l_{i}$ and $l_{j}$.

### SCCPMD method

#### Overview

**Figure 1:**
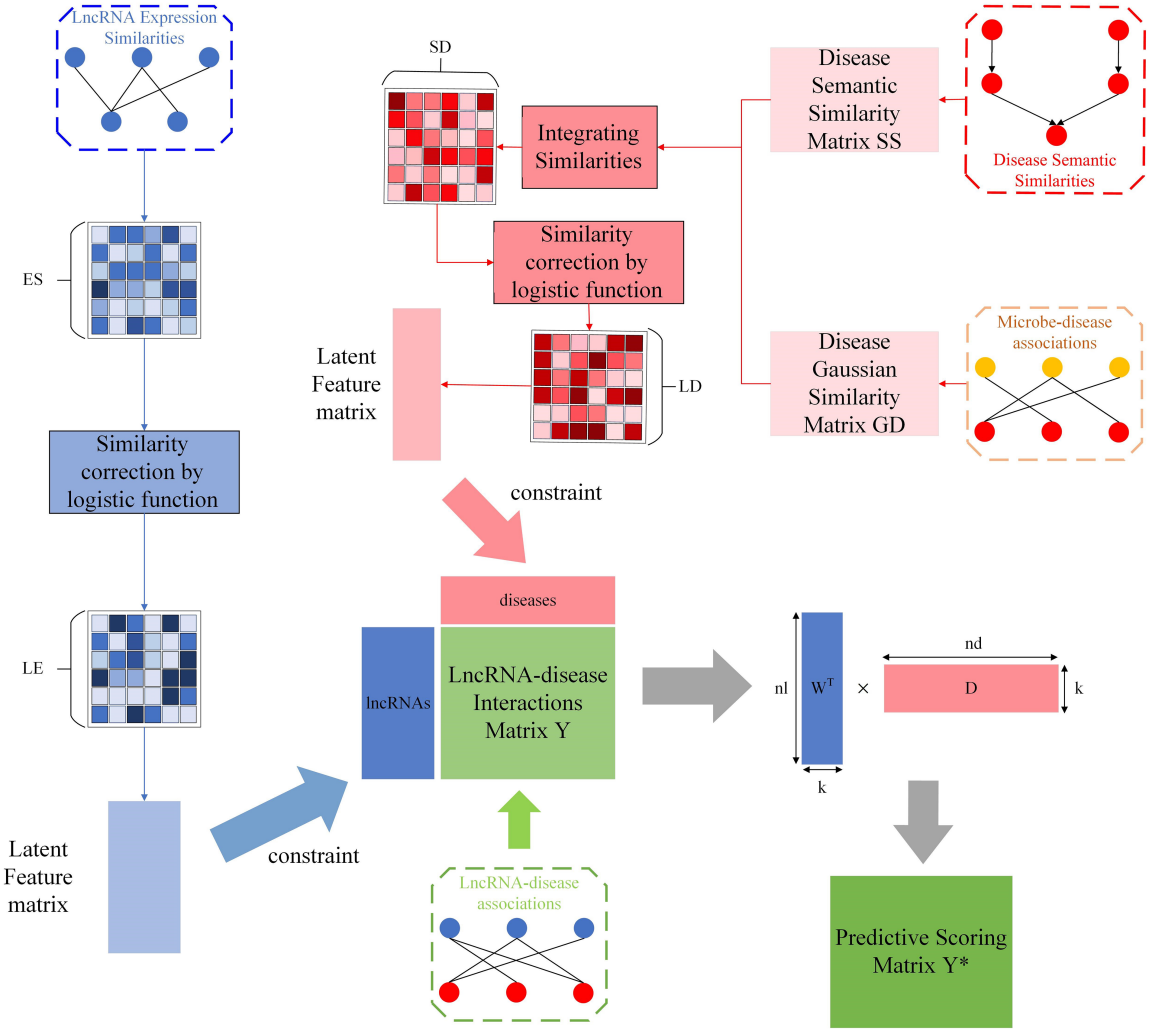
Flow chart of the SCCPMD approach.

SCCPMD involves the following five steps, which are schematically outlined in Figure 1: (i) constructing lncRNA–disease association networks, (ii) constructing DAGs based on MeSH information to calculate the disease semantic similarity SS and calculating disease Gaussian similarity GD based on microbe-disease associations, (iii) integration of disease semantic similarity and disease gaussian similarity to obtain disease similarity SD, (iv) calculating lncRNA expression similarity ES based on Spearman correlation coefficients, (v) performing logistic function transformation for similarity correction of disease similarity and lncRNA expression similarity to reduce the noise introduced by the similarity matrix during matrix decomposition, and (vi) using the proposed constrained probability matrix decomposition method to help predict potential lncRNA–disease associations.

#### Similarity correction

To reduce the noise that lncRNA and disease similarity matrices introduce during matrix decomposition, similarity correction techniques were used. The noise present in the similarity matrix is reduced by the logistic function so as to enhance the strong correlations in the similarity range [0,1] while diluting the weak correlations. This approach has previously been used in the study of disease-related genes (Vanunu et al., 2010). The logistic function is defined as follows:


(8) $$L\left(x\right)=\frac{1}{1+e^{ax+b}}$$

L(x)≈0 when x∈[0,0.3] and L(x)≈1 when x∈[0.6,1]. This means that weakly similar coefficients in the range of [0,0.3] are lost information, whereas strong similar coefficient values in the range of [0.6,1] usually exhibit significant co-expression of the relationship. Accordingly L(0) needs to be close to 0; therefore, we set L(0)=0.0001 to obtain b=log(9999). In addition, a is a correction degree coefficient that is used for parameter adjustment of the model. The corrected lncRNA expression similarity LE and the disease similarity LD are thus obtained as follows:


(9) $$LE\left(i,j\right)=\frac{1}{1+e^{a\times ES\left(i,j\right)+b}},\;\;i,j\in \left[1,nl\right]$$

(10) $$LD\left(i,j\right)=\frac{1}{1+e^{a\times DS\left(i,j\right)+b}},\;\;i,j\in \left[1,nd\right]$$

#### Constraint probability matrix decomposition

Following the similarity correction steps outlined above, we can obtain the association matrix Y representing the relationship between lncRNA and disease from the corrected lncRNA–lncRNA expression similarity LE and the corrected disease–disease similarity LD. The values of LE and LD fall in the [0,1] interval. Let $W\in R^{k\times nl}\;$and $D\in R^{k\times nd}$ be the lncRNA and disease latent feature matrices, where k∈min(nl,nd). The latent feature vectors specific to lncRNAs and diseases are represented by the column vectors $W_{i}$ and $D_{j}$, respectively. The goal is then to find lncRNA and disease latent models ($W\in R^{k\times nl}$ and $D\in R^{k\times nd}$) whose product ($W^{T}D$) can reconstruct the interaction matrix Y. From a probabilistic point of view, the conditional distribution of the observed interactions Y∈{0,1} is expressed as:


(11) $$P\left(Y|W,D,\sigma^{2}\right)=\overset{nl}{\underset{i=1}{\prod }}\overset{nd}{\underset{j=1}{\prod }}{\left[f\left(Y_{ij}|W_{i}^{T}D_{j},\sigma^{2}\right)\right]}^{I_{ij}}$$

where f(x|,μ|,σ2) is the probability density function of the Gaussian normal distribution with mean 𝜇 and variance $\sigma^{2}$, and $I_{ij}$ is the indicator function that is equal to 1 if the lncRNA$\;l_{i}$ is related with disease $d_{j}$ and is 0 otherwise. A probabilistic representation of the association matrix Y is then given by $P(Y|W,D,\sigma^{2}$). We use the following zero-mean spherical Gaussian priors on the lncRNA and disease eigenvectors as a generative model for the lncRNA and disease latent models:


(12) $$P\left(W|\sigma_{W}^{2}\right)=\overset{nl}{\underset{i=1}{\prod }}f\left(W_{i}|0,\sigma_{W}^{2}I\right)$$

(13) $$P\left(D|\sigma_{D}^{2}\right)=\overset{nd}{\underset{i=1}{\prod }}f\left(D_{j}|0,\sigma_{D}^{2}I\right)$$

where I is a k-dimensional identity diagonal matrix. Then, the posterior distribution of lncRNA and disease characteristics is derived as:


(14) $$\begin{array}{c} P\left(W,D|Y,\sigma^{2},\sigma_{W}^{2},\sigma_{D}^{2}\right)=\frac{P\left(W,D,Y,\sigma^{2},\sigma_{W}^{2},\sigma_{D}^{2}\right)}{P\left(Y,\sigma^{2},\sigma_{W}^{2},\sigma_{D}^{2}\right)}\\ =\frac{P\left(Y|W,D,Y,\sigma^{2}\right)\times P\left(W,D|\sigma_{W}^{2},\sigma_{D}^{2}\right)}{P\left(Y,\sigma^{2},\sigma_{W}^{2},\sigma_{D}^{2}\right)}\\ \sim P\left(Y|W,D,\sigma^{2}\right)\times P\left(W,D|\sigma_{W}^{2},\sigma_{D}^{2}\right)\\ =P\left(Y|W,D,\sigma^{2}\right)\times P\left(W|{\left(\sigma_{W}\right)}^{2}\right)\times P\left(D|\left(\sigma_{D}^{2}\right)\right)\\ =\overset{nl}{\underset{i=1}{\prod }}\overset{nd}{\underset{j=1}{\prod }}{\left[f\left(Y_{ij}|W_{i}^{T}D_{j},\sigma^{2}\right)\right]}^{I_{ij}}\\ \times \overset{nl}{\underset{i=1}{\prod }}f\left(W_{i}|0,\sigma_{W}^{2}I\right)\times \overset{nd}{\underset{i=1}{\prod }}f\left(D_{j}|0,\sigma_{D}^{2}I\right) \end{array}$$

Taking the logarithm of equation (11), the distribution is transformed to:


(15) $$\begin{array}{c} \ln P\left(W,D|Y,\sigma^{2},\sigma_{W}^{2},\sigma_{D}^{2}\right)=\frac{1}{2\sigma^{2}}\overset{nl}{\underset{i=1}{\sum }}\overset{nd}{\underset{j=1}{\sum }}I_{ij}{\left(Y_{ij}-W_{i}^{T}D_{j}\right)}^{2}\\ -\frac{1}{2\sigma^{2}}\overset{nl}{\underset{i=1}{\sum }}W_{i}^{T}W_{i}-\frac{1}{2\sigma^{2}}\overset{nd}{\underset{j=1}{\sum }}D_{j}^{T}D_{j}\\ -\frac{1}{2}\left(\begin{array}{l} \left(\overset{nl}{\underset{i=1}{\sum }}\overset{nd}{\underset{j=1}{\sum }}I_{ij}\right)\ln\sigma^{2}\\ +\left(nl\right)k\ln\sigma_{W}^{2}+\left(nd\right)k\ln\sigma_{D}^{2} \end{array}\right)+c \end{array}$$

where c is a constant. With the hyperparameters held constant, maximization of the log posterior for lncRNA and disease characteristics is identical to minimization of the sum of squared errors with a quadratic regularization term objective function:


(16) $$\min\frac{1}{2}\overset{nl}{\underset{i=1}{\sum }}\overset{nd}{\underset{j=1}{\sum }}I_{ij}{\left(Y_{ij}-W_{i}^{T}D_{j}\right)}^{2}+\frac{\lambda_{W}}{2}\overset{nl}{\underset{i=1}{\sum }}{\left\|W_{i}\right\|}_{Fro}^{2}+\frac{\lambda_{D}}{2}\overset{nd}{\underset{j=1}{\sum }}{\left\|D_{j}\right\|}_{Fro}^{2}$$

where $\lambda_{W}=\sigma^{2}/\sigma_{W}^{2}$，$\lambda_{D}=\sigma^{2}/\sigma_{D}^{2}$, ${\left\|\cdot \right\|}_{Fro}^{2}$ represents the Frobenius norm. However, the conventional probabilistic matrix decomposition model only uses a probabilistic linear model with Gaussian noise to depict the interaction between lncRNAs and diseases, leaving room for improvement. Based on the assumption that similar lncRNAs are usually interrelated with corresponding diseases and vice versa, CPMD takes more biological information (such as the similarity of lncRNAs and diseases) into account for the prediction. Accordingly, we suggest the following as a new objective function for CPMD:


(17) $$\begin{array}{l} \min\frac{1}{2}\overset{nl}{\underset{i=1}{\sum }}\overset{nd}{\underset{j=1}{\sum }}I_{ij}{\left(Y_{ij}-W_{i}^{T}D_{j}\right)}^{2}+\frac{\lambda_{W}}{2}\overset{nl}{\underset{i=1}{\sum }}{\left\|W_{i}\right\|}_{Fro}^{2}+\frac{\lambda_{D}}{2}\overset{nd}{\underset{j=1}{\sum }}{\left\|D_{j}\right\|}_{Fro}^{2}\\ +\frac{\lambda_{1}}{2}{\left\|W^{T}W-LD\right\|}_{Fro}^{2}+\frac{\lambda_{2}}{2}{\left\|D^{T}D-LE\right\|}_{Fro}^{2} \end{array}$$

where $W_{i}$ represents the k-dimensional potential feature vector of lncRNAs, $W^{T}W\;$is the lncRNA weighted similarity matrix, and $D^{T}D$ is the disease weighted similarity matrix. Here, we use the gradient descent algorithm to solve the optimization problem in equation (14). First, the corresponding Lagrangian function $\Gamma_{f}$ of equation (14) is defined as:


(18) $$\begin{array}{c} \Gamma_{f}=\frac{1}{2}Tr\left(I\times \left(YY^{T}-YD^{T}W-W^{T}DY^{T}+W^{T}DD^{T}W\right)\right)\\ +\frac{\lambda_{W}}{2}Tr\left(WW^{T}\right)+\frac{\lambda_{D}}{2}Tr\left(DD^{T}\right)\\ +\frac{\lambda_{1}}{2}Tr\left(LD{\left(LD\right)}^{T}-LDW^{T}W-W^{T}W\left(LD\right)+W^{T}WW^{T}W\right)\\ +\frac{\lambda_{2}}{2}Tr\left(LE{\left(LE\right)}^{T}-LED^{T}D-D^{T}D\left(LE\right)+D^{T}DD^{T}D\right)\\ +Tr\left(\Phi W^{T}+Tr\left(\Psi D^{T}\right)\right) \end{array}$$

where 𝑇𝑟(∙) denotes the trace of the matrix, and 𝛷=[$\varphi_{ik}$] and 𝛹=[$\psi_{jk}$] are the constraints 𝑊_𝑖𝑘_≥0 and D_𝑗𝑘_≥0 for Lagrange multipliers. The partial derivatives of W and D are:


(19) $$\begin{array}{c} \frac{\partial \Gamma_{f}}{\partial W}=I\times \left(-DY^{T}+DD^{T}W\right)\\ +\lambda_{W}W+2\lambda_{1}\left(-W\left(LD\right)+WW^{T}W\right)+\Phi \end{array}$$

(20) $$\begin{array}{c} \frac{\partial \Gamma_{f}}{\partial D}=I\times \left(-WY^{T}+WW^{T}W\right)+\lambda_{D}D\\ +2\lambda_{2}\left(-D\left(LE\right)+DD^{T}D\right)+\Psi \end{array}$$

Using the Karush-Kuhn-Tucker conditions $\varphi_{ik}W_{ik}$=0 and $\psi_{jk}D_{jk}$=0，the following equations for $W_{ik}$ and $D_{jk}$ can be obtained:


(21) $$\begin{array}{l} {\left(I\times \left(-DY^{T}+DD^{T}W\right)\right)}_{ik}W_{ik}+{\left(\lambda_{W}W\right)}_{ik}W_{ik}\\ +{\left(2\lambda_{1}\left(-W\left(LD\right)+WW^{T}W\right)\right)}_{ik}W_{ik}=0 \end{array}$$

(22) $$\begin{array}{l} {\left(I\times \left(-WY+WW^{T}D\right)\right)}_{jk}D_{jk}+{\left(\lambda_{D}D\right)}_{jk}D_{jk}\\ +{\left(2\lambda_{2}\left(-D\left(LE\right)+DD^{T}D\right)\right)}_{jk}D_{jk}=0 \end{array}$$

Thus, we can obtain the following update rule:


(23) $$W_{ik}\times \frac{{\left(I\times \left(DY^{T}\right)+2\lambda_{1}\left(W\left(LD\right)\right)\right)}_{ik}}{{\left(I\times \left(DD^{T}W\right)\right)}_{ik}+{\left(\lambda_{W}W\right)}_{ik}+{\left(2\lambda_{1}\left(WW^{T}W\right)\right)}_{ik}}\to W_{ik}^{new}$$

(24) $$D_{jk}\times \frac{{\left(I\times \left(WY\right)+2\lambda_{2}\left(D\left(LE\right)\right)\right)}_{jk}}{{\left(I\times \left(WW^{T}D\right)\right)}_{jk}+{\left(\lambda_{D}D\right)}_{jk}+{\left(2\lambda_{2}\left(DD^{T}D\right)\right)}_{jk}}\to D_{jk}^{new}$$

In accordance with equations (20) and (21), the matrices W and D are continuously updated until reaching the objective function’s local minimum. Finally, the predicted lncRNA–disease interaction matrix is calculated using the formula $Y^{*}=W^{T}D$. In general, the 𝑗th column of $Y^{*}$ indicates the interaction score between disease $d_{j}$ and the lncRNA, with a higher score indicating a more significant interaction.

## Results and discussion

### Assessment indicators

Both LOOCV and 5-fold CV methods were utilized to assess the SCCPMD model’s efficacy in predicting potential lncRNA–disease associations (Huang et al., 2022c; Sun et al., 2022). Each proven lncRNA–disease association is listed as a test sample in the LOOCV framework, whereas the other unidentified relationship pairings are listed as training samples. All confirmed lncRNA–disease associations are separated into five groups in the 5-fold CV framework, and in each experiment, one group is chosen as the test group and the other as the training group. Using this method, we ran the experiment 100 times and computed the mean of all outcomes. Since the lncRNA–disease dataset only contains a small number of known lncRNA–disease associations and the AUC is known to be insensitive to a skewed class distribution, we used the AUC of the receiver operating characteristic curve to evaluate the performance of SCCPMD (Zhao et al., 2022).

### Optimal parameter selection

**Figure 2:**
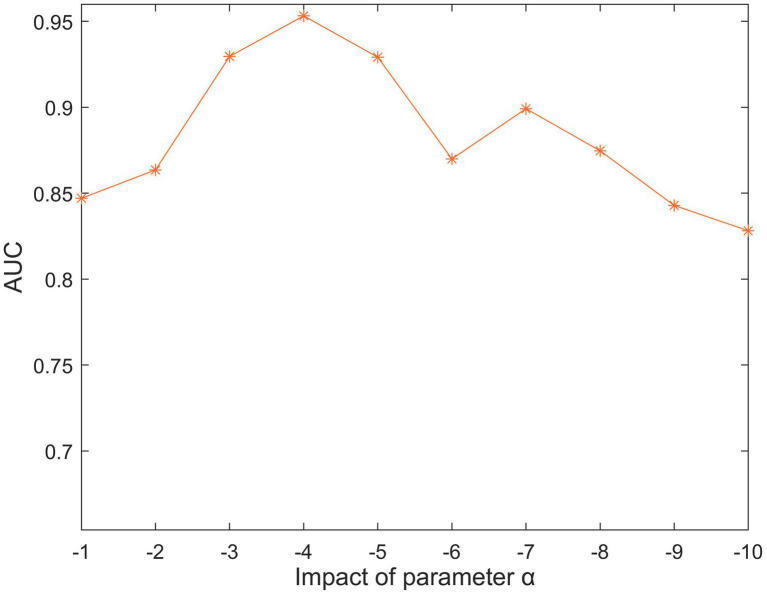
The impact of different α values under 5-fold cross-validation.

There are six parameters in SCCPMD: $a,k,\lambda_{W},\lambda_{D},\lambda_{1}$, and $\lambda_{2}$. To tease out the effect of these five parameter choices on the model, we performed 100 experiments in the 5-fold CV framework and calculated the average AUC values. First, there is a similarity correction component for parameter a. We searched for the optimal parameter in the range of −1 to −10. Figure 2 clearly shows that the highest AUC value was reached when a=−4.

**Figure 3:**
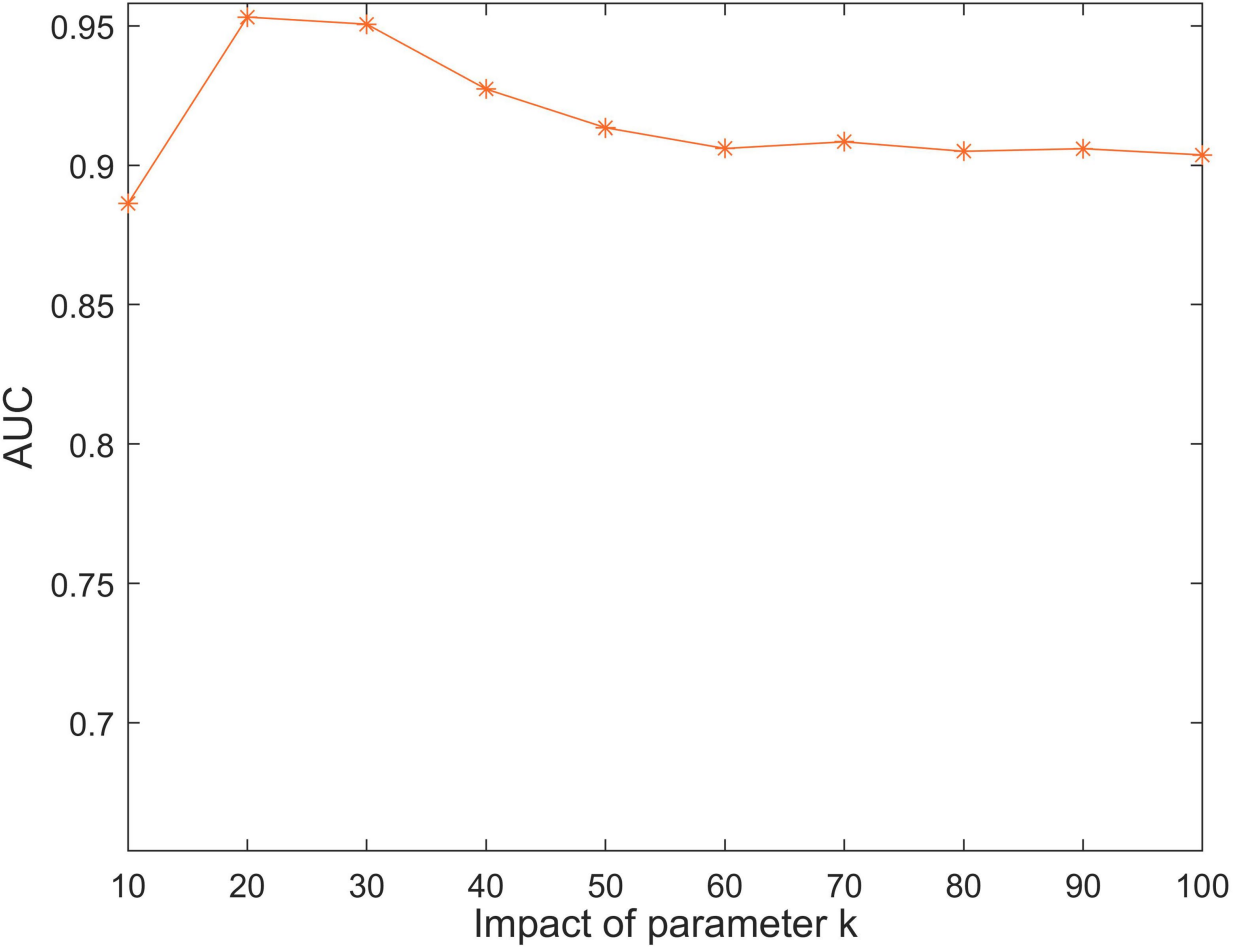
The impact of different k values under 5-fold cross-validation.

The parameter k represents the number of lncRNA and disease latent feature matrix row vectors, which determines the size of the latent feature matrix. As shown in Figure 3, we restricted the range of k from 10 to 100. The highest AUC value was achieved for SCCPMD whenk=20.

**Figure 4:**
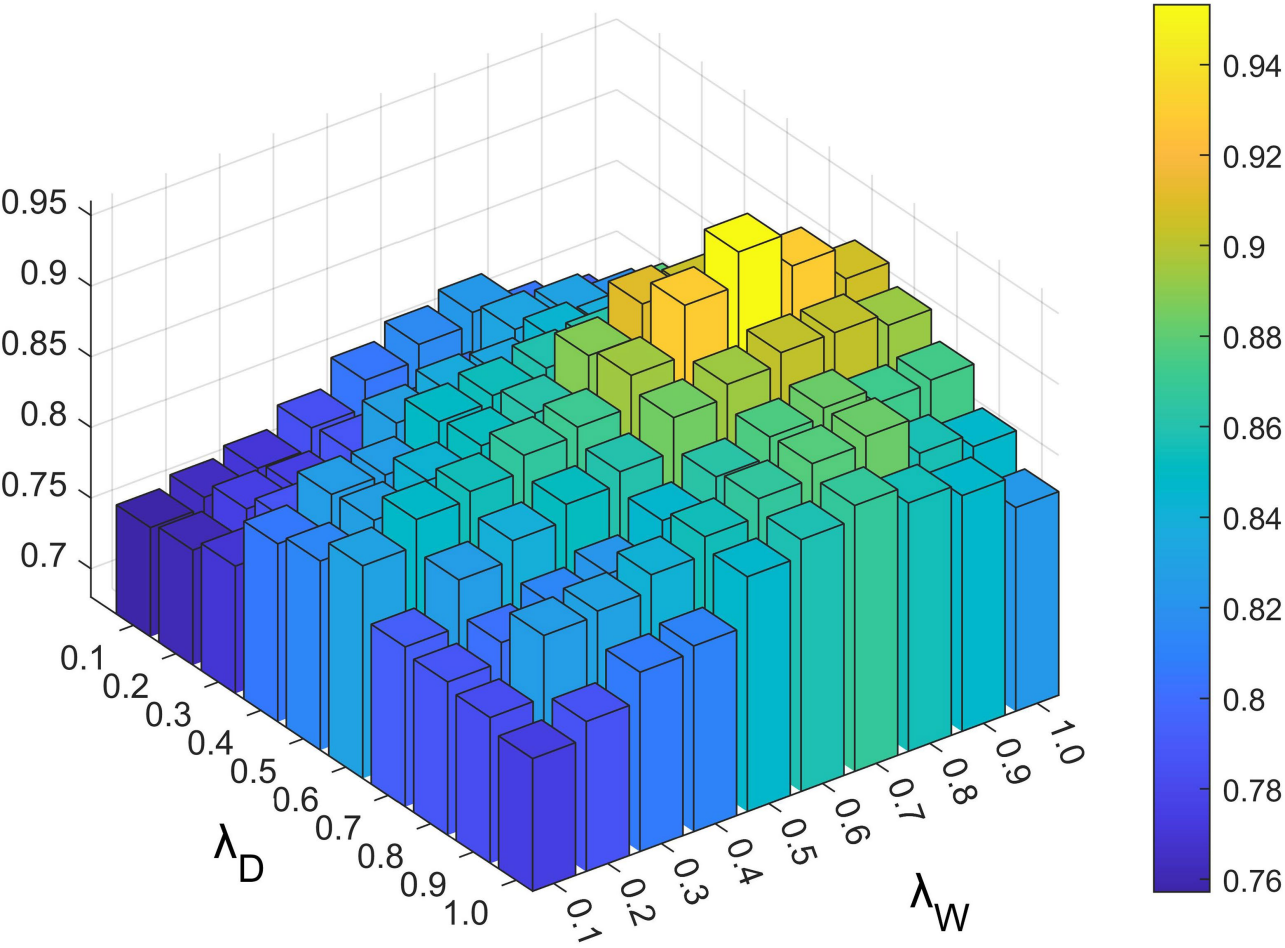
The impact of different $\lambda_{W}$ and $\lambda_{D}$ values under 5-fold cross-validation.

**Figure 5:**
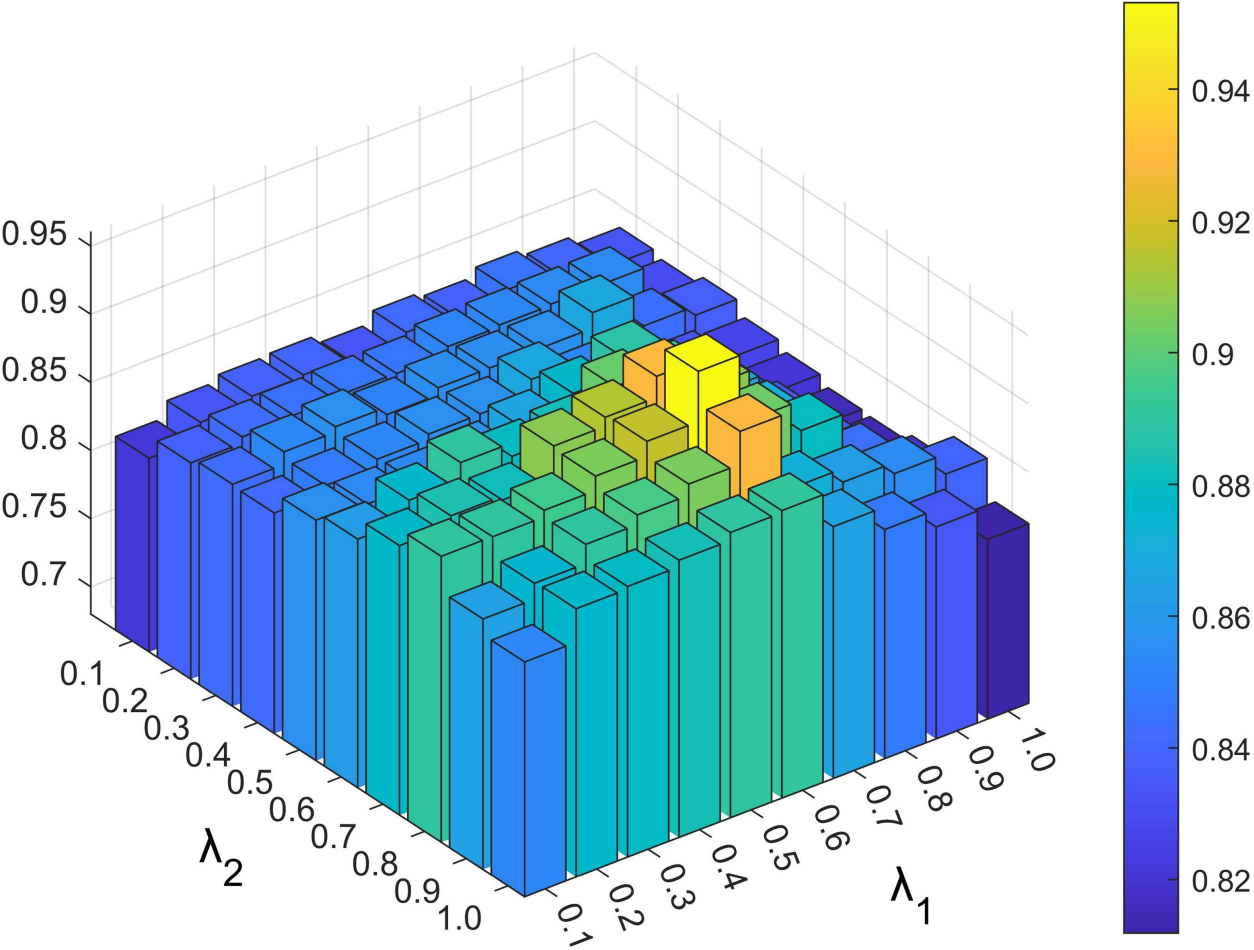
The impact of different $\lambda_{1}$ and $\lambda_{2}$ values under 5-fold cross-validation.

Parameters $\lambda_{W},\lambda_{D},\lambda_{1},$and $\lambda_{2}\;$exist in the constrained probability matrix decomposition part, which controls the influence of each part in the final update rule of the lncRNA and disease characteristic matrix. As shown in Figures 4, 5, we set the range of all four parameters to be from 0.1 to 1.

Based on the above experiments, the best values of these five parameters were finally determined as $a=-4,k=20,\lambda_{W}=0.8,\lambda_{D}=0.6,\lambda_{1}=0.6$, and $\lambda_{2}=0.8$.

### Algorithm comparison

**Figure 6:**
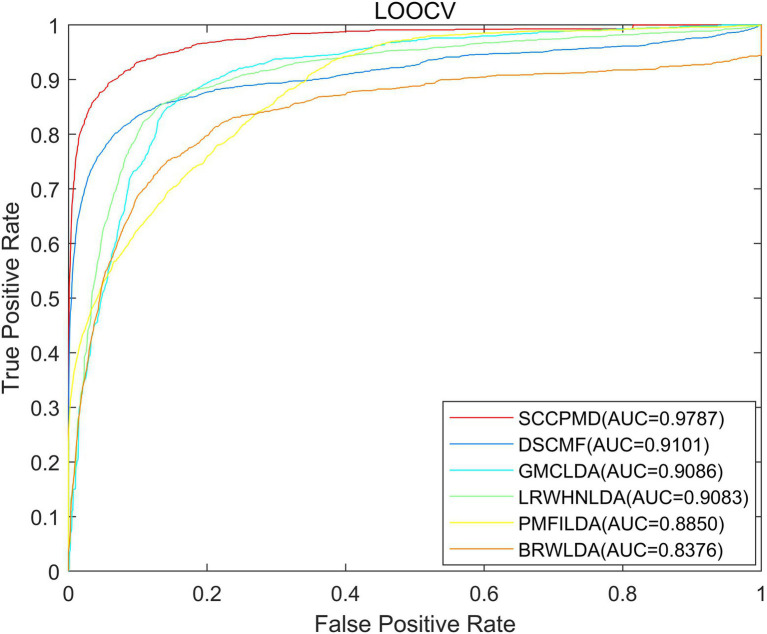
Area under the receiver operating characteristic curve (AUC) values of leave-one-out cross-validation (LOOCV) between SCCPMD and the other five comparison models.

**Figure 7:**
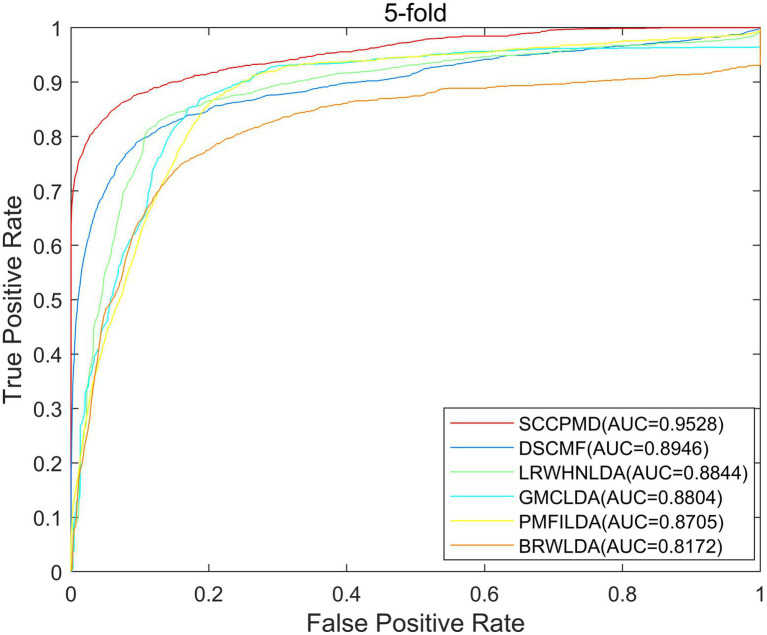
Area under the receiver operating characteristic curve (AUC) values of 5-fold cross-validation between SCCPMD and the other five comparison models.

To evaluate the predictive performance of the SCCPMD model, SCCPMD was compared with five existing advanced methods: dual sparse collaborative matrix factorization (DSCMF; Liu et al., 2021), geometric matrix completion lncRNA–disease association (GMCLDA; Lu et al., 2020), local random walk-based prediction of human lncRNA and disease associations (Li et al., 2021), probabilistic matrix factorization method for identifying lncRNA–disease associations (PMFILDA; Xuan et al., 2019), and bi-random walks for predicting lncRNA–disease associations (BRWLDA; Yu et al., 2017). As shown in Figure 6, the AUC value of the SCCPMD curve in the LOOCV framework was 0.9787, which was larger than that obtained with the other prediction methods (DSCMF, AUC = 0.9101; GMCLDA, AUC = 0.9086; LRWHNLDA, AUC = 0.9083; PMFILDA, AUC = 0.8850; and BRWLDA, AUC = 0.8376), indicating that the performance of SCCPMD is better than that of existing calculation methods. To further validate the prediction performance of SCCPMD, the 5-fold CV framework was used for validation. As shown in Figure 7, SCCPMD obtained a reliable AUC of 0.9528 ± 0.0036, which was much higher than the AUC values of 0.8946 ± 0.0038, 0.8804 ± 0.0009, 0.8844 ± 0.0014, 0.8705 ± 0.0047, and 0.8172 ± 0.0014 for the comparison methods DSCMF, GMCLDA, LRWHNLDA, PMFILDA, and BRWLDA, respectively. The computational methods we compared were only for lncRNA-disease association pairs, predicting potential associations based on the similarity between lncRNA and disease. The SCCPMD model uses microbe-disease associations to enrich disease similarities, while correcting the similarity matrix to highlight strong similarities and reduce noise in the original similarities. Therefore, SCCPMD shows better performance than these five methods and would be more favorable for the prediction of lncRNA–disease associations.

### Case study

Malignancy, as a general term to refer to cancer, has a significant negative impact on human health. With a global annual mortality rate of more than 10 million, cancer remains one of the main contributors to mortality (Zaimy et al., 2017). To validate the actual predictive performance of SCCPMD for lncRNA–disease associations, three cancer types with high hazard were selected as disease case studies: breast cancer, lung cancer, and RCC. The predicted correlations were validated in three lncRNA–disease association databases: the lncRNA disease database, Lnc2cancer database, and MNDR database.

Table 1 shows the top 10 lncRNAs that were predicted to be associated with breast cancer using our model, nine of which have previously been reported to be associated with breast cancer. Breast epithelial cells can become cancerous when they proliferate uncontrollably in response to several oncogenic stimuli (Fahad, 2019). Four lncRNAs, including *LINC00667*, were identified by analysis of gene expression data from 768 breast cancer patients in The Cancer Genome Atlas database, suggesting potential predictive biomarkers for breast cancer with clinical value (Zhu et al., 2020). Among these markers, *PVT1* has been reported to affect mature adipogenic mediators by regulating p21 expression in triple-negative breast cancer cells (Wang et al., 2018b). Functional studies showed that the proliferation, migration, and invasion of breast cancer cells overexpressing *LINC01089* were significantly reduced and that epidermal growth factor reversed these effects (Yuan et al., 2019). *TSIX* is an lncRNA that has been explored as a stable non-invasive breast cancer immunological biomarker, which plays a role in X chromosome inactivation and breast cancer (Salama et al., 2020).

**Table 1:** Top 10 lncRNAs predicted by SCCPMD to be connected to breast cancer.

Rank	lncRNA name	Evidence (PubMed ID)
1	*LINC00667*	31,897,133
2	*PVT1*	30,371,726
3	*PINK1-AS*	unknown
4	*LINC01089*	31,417,284
5	*TSIX*	31,998,636
6	*MSR1*	26,967,566
7	*LINC01638*	30,002,443
8	*CDKN2B-AS1*	unknown
9	*H19*	32,124,962
10	*NEAT1*	30,957,286

Table 2 shows the top 10 lncRNAs that were predicted to be associated with lung cancer using our model, all of which have been reported to play roles in lung cancer. Despite improvements in our knowledge of lung cancer risk, progression, immunologic control, and treatment choices, lung cancer—a malignancy that starts in the bronchial mucosa or glands of the lungs—remains the most common cause of cancer-related death (Bade and Cruz, 2020). Amplification of *PVT1* in lung cancer patients was associated with a poor prognosis for survival. *PVT1* levels are increased in lung cancer cells, which promotes their growth and metastasis both *in vivo* and *in vitro* (Pan et al., 2020). The expression of *SNHG1* in non-small cell lung cancer (NSCLC) tissues and cells is high. Silencing *SNHG1* could suppress the migration and invasion of NSCLC cells, which also promoted apoptosis and decreased the cell proliferation rate (Li and Zheng, 2020). Considerable upregulation of the lncRNA *CDKN2 B-AS1* has been detected in both lung cancer tissues and cell lines (Wang et al., 2020). *In vitro* studies demonstrated that blocking *NEAT1* with short hairpin RNA prevented lung cancer cells from surviving and migrating or invading (Ma et al., 2020). Table 3 shows the top 10 lncRNAs that were predicted to be associated with RCC with our model, all of which have been associated with RCC in previous studies. RCC comprises a group of malignant tumors originating from the renal cortical epithelium, most commonly in the upper pole of the kidney (Pullen Jr, 2021). By inhibiting cell cycle progression and reversing the epithelial-to-mesenchymal transition (EMT) phenotype, *NEAT1* knockdown could reduce the rate of RCC cell proliferation and suppressed RCC migration and invasion (Liu et al., 2017). By controlling EMT-related genes, loss-of-function and gain-of-function pathways demonstrated that *CRNDE* promotes the migration and invasion of clear cell RCC cells (Ding et al., 2018). *MEG3* has been proposed to induce apoptosis in RCC cells by activating the mitochondrial pathway (Wang et al., 2015). Functional assays revealed that *MIAT* knockdown prevented kidney cancer cells from proliferating and metastasizing both *in vitro* and *in vivo* (Qu et al., 2018).

**Table 2:** Top 10 lncRNAs predicted by SCCPMD to be connected to lung cancer.

Rank	lncRNA name	Evidence (PubMed ID)
1	*PVT1*	33,167,678
2	*SNHG1*	31,788,970, 28,147,312
3	*CDKN2B-AS1*	33,116,641
4	*NEAT1*	32,296,457, 31,646,570
5	*MEG8*	30,262,664
6	*KCNQ1OT1*	31,486,494
7	*MALAT1*	32,141,554
8	*H19*	31,190,899
9	*MEG3*	31,585,300
10	*PCAT6*	30,464,520

**Table 3:** Top 10 lncRNAs predicted by SCCPMD to be connected to renal cell carcinoma.

Rank	lncRNA name	Evidence (PubMed ID)
1	*NEAT1*	28,968,960
2	*CRNDE*	30,129,055
3	*MEG3*	26,223,924
4	*MIAT*	30,041,179
5	*PVT1*	31,040,699, 29,725,470
6	*SNHG5*	32,281,285, 32,194,910
7	*HOTAIRM1*	31,862,408
8	*MEG3*	31,071,531
9	*TUG1*	31,310,753
10	*ZFAS1*	30,841,471

## Conclusion

An increasing number of studies have shown that exploration of potential lncRNA–disease associations can be expedited and more effectively performed by developing computational models. Recent results have also showed that matrix decomposition is a reliable method for predicting lncRNA-disease associations. We here propose a novel method to predict unknown lncRNA–disease associations based on corrected similarity added as a constraint to the probability matrix decomposition (SCCPMD). We confirmed the excellent performance of SCCPMD, demonstrating superiority in prediction to existing advanced algorithms, which is attributed to the following three factors: (1) the disease Gaussian similarity obtained by fusing microbe-disease associations calculation can solve the original problem of sparse disease semantic similarity, (2) the corrected similarity performance highlights the effects of strong correlations while reducing the effects of weak correlations, thus reducing the overall noise in the matrix; and (3) introducing lncRNA and disease similarity constraints in the traditional probability matrix decomposition makes better use of this biological information to improve the prediction performance. The AUC values of SCCPMD in the LOOCV and 5-fold CV frameworks reached up to 0.9787 and 0.9528 ± 0.0036, respectively, which were much higher than those obtained with the comparative algorithms. Additionally, we chose three complex diseases as case studies, demonstrating that SCCPMD performs well with real-world clinical data.

Although SCCPMD enriches disease similarity using microbe-disease associations, prediction results are also affected by microbe-disease associations. In addition, relying on a single lncRNA expression similarity can also make the model limited. Integration of more similarity information is expected to make the proposed model more robust. Therefore, in future work we will try to combine more bioinformatic datasets and fuse multiple lncRNA similarities to improve the robustness and predictive performance of the model.

## Data availability statement

The datasets presented in this study can be found in online repositories. The names of the repository/repositories and accession number(s) can be found in the article/supplementary material.

## Ethics statement

Ethical review and approval were not required for the study of human participants in accordance with the local legislation and institutional requirements. Written informed consent from the patients/ participants OR patients/participants legal guardian/next of kin was not required to participate in this study in accordance with the national legislation and the institutional requirements.

## Author contributions

LL, HJ, and LC: conceptualization. LL: data curation and resources. LL, RC, YZ, WX, HJ, LC, and MZ: formal analysis and writing—review and editing. LL, RC, and YZ: investigation. LL, RC, YZ, WX, HJ, and LC: methodology and supervision. LL and MZ: project administration. RC, YZ, and WX: validation and writing draft. RC: visualization. All authors contributed to the article and approved the submitted version.

## Funding

This work was supported by the National Natural Science Foundation of China (72001202 and 62002070), the Opening Project of Guangdong Province Key Laboratory of Computational Science at Sun Yat-sen University (2021013), the Science and Technology Plan Project of Guangzhou City (202102021236), and the Philosophy and Social Science Co-Construction Project of Guangzhou City (2020GZGJ115).

## Conflict of interest

The authors declare that the research was conducted in the absence of any commercial or financial relationships that could be construed as a potential conflict of interest.

## Publisher’s note

All claims expressed in this article are solely those of the authors and do not necessarily represent those of their affiliated organizations, or those of the publisher, the editors and the reviewers. Any product that may be evaluated in this article, or claim that may be made by its manufacturer, is not guaranteed or endorsed by the publisher.

## Footnotes

^1^ https://www.nlm.nih.gov/mesh/meshhome.html
